# Genomic characterization of antimicrobial resistance and virulence determinants in *Salmonella* Infantis isolated from human, food, and animal sources

**DOI:** 10.1128/aem.01975-25

**Published:** 2026-03-23

**Authors:** Jing Han, Eric Tang, Shaohua Zhao, Steven L. Foley

**Affiliations:** 1 Division of Microbiology, FDA National Center for Toxicological Research, Jefferson, Arkansas, USA; 2 Little Rock Central High School 248533 , Little Rock, Arkansas, USA; 3 Office of Applied Science, FDA Center for Veterinary Medicine, Laurel, Maryland, USA; Centers for Disease Control and Prevention, Atlanta, Georgia, USA

**Keywords:** *Salmonella *serovar Infantis, antimicrobial resistance, virulence, plasmid transfer

## Abstract

*Salmonella* Infantis has emerged as a significant cause of foodborne illness, largely due to its increasing antimicrobial resistance (AMR) and capacity to cause widespread disease. A key contributor to its emergence is the acquisition of the pESI plasmid, which harbors multiple virulence genes and resistance determinants to multiple antimicrobial classes. This enhances the spread of multidrug-resistant strains, complicating treatment and increasing infection risks. To understand the genetic factors contributing to the growing prevalence of *S*. Infantis, whole genome sequencing (WGS) of 15,806 isolates from various sources was analyzed. WGS data extracted from GenBank and EnteroBase were assessed for their putative virulence genes and plasmid transfer genes using the Virulence and Plasmid Transfer Factor Database. Metadata and AMR gene profiles were extracted from the NCBI Pathogen Detection Isolate Browser or analyzed using AMRFinderPlus when unavailable in NCBI. A total of 153 distinct AMR genes were detected, with 68.24% (*N* = 10,786) of the isolates carrying at least one AMR gene. The most observed resistance genes included *tet(A)* (*N* = 9,385; 59.38%), *sul1* (*N* = 9,177; 58.06%), *aadA1* (*N* = 9,026; 57.10%). Notably, 66.27% (*N* = 10,474) of isolates carried at least one transfer gene associated with IncI1. The six pESI plasmids examined in this study contained all IncI1-related transfer genes except *IncI1_pilI* and *IncI1_traD*, which suggested the possible recombination or mosaicism between IncI1- and pESI-like elements. Moreover, most of the isolates (87.45%; *N* = 13,822) shared a common virulence gene profile containing 435 out of the 594 putative virulence genes examined, with only minor variations among the remaining isolates. The considerable overlap in virulence genes, AMR genes, and plasmid transfer gene profiles between human isolates and those from other sources implies potential links between foodborne contamination and human infections.

**IMPORTANCE:**

This study provides a comprehensive genomic analysis of over 15,000 *Salmonella* Infantis isolates, revealing widespread antimicrobial resistance (AMR), virulence gene prevalence, and plasmid transfer potential. The high frequency of key AMR genes and the detection of chimeric plasmids highlight the evolving threat posed by multidrug-resistant *S.* Infantis. The strong similarity in virulence gene profiles between human and non-human isolates underscores the potential for zoonotic transmission through the food chain. These findings offer crucial insights into the mechanisms driving the emergence and spread of resistant *S.* Infantis, informing future surveillance, risk assessment, and control strategies to protect public health.

## INTRODUCTION

*Salmonella enterica* is a major global foodborne pathogen, responsible for an estimated 94 million infections annually and approximately 215,000 deaths per year (1–3). Among the more than 2,600 serovars identified for *S. enterica*, *S. enterica* serovar Infantis (*S*. Infantis) has recently received much attention due to its increasing antimicrobial resistance (AMR) and its ability to cause widespread disease. *S*. Infantis is frequently isolated from various animal hosts and animal-derived food products (1, 4), and it has emerged as one of the top 10 serovars causing human salmonellosis in the United States, significantly impacting public health (4, 5). In fact, both the U.S. Foodborne Diseases Active Surveillance Network (FoodNet) and CDC BEAM dashboard have reported a notable increase in *S*. Infantis since 2014 (6, 7).

A distinctive feature of many recently isolated *S*. Infantis strains is their possession of pESI (plasmid for emergent *Salmonella* Infantis), which was first reported in Israel in 2014 (8). Since then, pESI-positive *S*. Infantis strains have been detected worldwide, including the Americas, Europe, Western Pacific, Eastern Mediterranean, Africa, and Asia (1, 8–10). The pESI is an approximately 280 kb, self-transferred, mosaic megaplasmid (6, 8, 11, 12) that encodes multiple AMR genes, including those for β-lactams (e.g., *bla*_CTX-M-1_, *bla*_CTX-M-65_, and *bla*_TEM-1_), streptomycin and spectinomycin (*aadA1*), sulfonamides (*sul1*), tetracycline [*tet(A)*], and trimethoprim (*dfrA*). Additionally, pESI harbors multiple virulence factors (VFs), such as the yersiniabactin siderophore cluster (*ybt*, *fyuA*, and *irp12*) for iron acquisition during infection and two fimbria clusters (chaperone-usher fimbria cluster and the Infantis plasmid-encoded fimbria cluster) that may promote biofilm formation and epithelial adhesion (13). Additionally, pESI harbors a *mer* operon, enhancing bacterial tolerance to environmental mercury and broadening adaptive capabilities in diverse environments. Using its toxin-antitoxin-based killing mechanisms, the plasmid also exhibits remarkable stability and transferability, facilitating its dissemination among bacterial populations, posing significant challenges for clinical management and infection control (11, 14).

Several studies have linked the rapid emergence of *S*. Infantis to the presence of pESI or pESI-like megaplasmids (11, 12, 15). In addition to *S*. Infantis, pESI-like megaplasmids have also been identified in other *Salmonella* serotypes, including Agona, Muenchen, Schwarzengrund, and Senftenberg (11). The ability of *Salmonella* to easily acquire pESI raises significant concerns for health and food safety. The intricate interplay between AMR and virulence encoded within pESI underscores the urgent need for enhanced surveillance and development of novel therapeutic strategies to combat infections caused by multidrug-resistant (MDR) *S*. Infantis.

AMR among *Salmonella* strains, including *S*. Infantis, poses a substantial challenge in the management and treatment of infections, leading to higher morbidity and mortality rates (16, 17). Given the rapid spread of the AMR across multiple antimicrobial classes, it is imperative to develop innovative strategies for controlling bacterial pathogens and treating bacterial infectious diseases. Despite the recent rise in reported cases and its unique AMR traits, comprehensive data on the virulence profiles of *S*. Infantis remain scarce. Therefore, this study aims to assess the genetic diversity of S. Infantis with particular focus on virulence factors, AMR genes, and plasmid contents through an extensive analysis of whole genome sequence (WGS) data from isolates collected from diverse sources, geographic locations, and time frames (1980–2024) available in the GenBank database and EnteroBase.

## MATERIALS AND METHODS

### Bacterial strains analyzed and data extraction

To analyze the genetic characteristics of *S*. Infantis, all publicly available WGS data—including assemblies at different levels: (i) contig, (ii) scaffold, (iii) chromosome, and (iv) complete—were downloaded from NCBI. Data included sequences released between 1980 (the earliest available) and 12 August 2024. Metadata, including isolate source/type, geographic location, year of isolation (if available), and SNP types, were extracted from GenBank and the NCBI Pathogen Detection Isolate Browser (https://www.ncbi.nlm.nih.gov/pathogens/). The available genome quality control metrics, including genome size, number of contigs, N50 value, and total gene count, were also extracted from NCBI assembly records to enhance the completeness and reliability of the genomic data. Also, all publicly available WGS and metadata of *S*. Infantis from EnteroBase were downloaded on 5 December 2025. The metadata were recorded in a spreadsheet for further analysis.

### Virulence, plasmid, and AMR gene detection

To predict putative virulence genes and plasmid transfer genes, WGS FASTA files for each strain were uploaded to the *Salmonella* Virulence Factor and Plasmid Transfer Gene Comparison Tools (https://virulence.fda.gov) (18, 19). An e-value cutoff of 10^−3^ was automatically applied when using these tools (18), consistent with the threshold used in NCBI BLAST searching (https://biopython.org/docs/1.76/api/Bio.Blast.Applications.html). Genes with an e-value below 10^−3^ were considered present in the WGS and are included in the assessment result. AMR genes of the isolates from NCBI were extracted directly from the metadata columns in the NCBI Pathogen Detection Isolate Browser (20), while the WGS FASTA files of the isolates from EnteroBase were analyzed with AMRFinderPlus (ver. 26.1) within the GalaxyTrakr platform (21). The presence or absence of predicted virulence genes, plasmid transfer genes, and AMR genes was recorded as binary values (1 = present, 0 = absent). To assess whether AMR rates varied among countries, rates were calculated only for countries with more than 100 isolates to minimize the impact of small sample sizes and enable meaningful cross-country comparisons.

### pESI plasmid analysis

To compare detected plasmids with pESI plasmids, sequences of pESI plasmids from five *S*. Infantis isolates (13065790-183 [GenBank accession no. NZ_OW849855.1], 14026835 [GenBank accession no. NZ_OW849859.1], 119944 [GenBank accession no. NZ_CP047882.1], MRS17_00712 [GenBank accession no. CP103792.1], and 13065790-183 [GenBank accession no. OW849855.1]), as well as one pESI from serovar Muenchen strain 180135033 (GenBank accession no. NZ_CP088902.1), were randomly selected and downloaded from NCBI. These plasmids were selected because they have complete, fully assembled sequences available in GenBank and have been previously characterized and confirmed as pESI plasmids. All selected plasmid sequences were analyzed using the Plasmid Transfer Gene Profile Assessment Tool to assess the incompatibility group (Inc)-associated plasmid transfer genes, along with the *Salmonella* and *Escherichia coli* Virulence Factor Assessment tools to identify the virulence genes they contain.

### Data analyses

Binary data were entered into Excel (Microsoft, Redlands, WA, USA) and formatted for analyses in BioNumerics (ver. 7.6; Applied Maths, Austin, TX, USA). As described previously (22), descriptive statistics on the prevalence of AMR, potential virulence factors, and plasmid transfer-associated genes were generated in Excel. Phylogenetic analyses were conducted using BioNumerics based on the presence or absence of genes across different metadata categories. The analyses performed in BioNumerics included the identification of polymorphic characters (defined here as genes present in between 0.5% and 99.5% of strains), principal component analysis, and the calculation of minimal spanning trees based on the comparison of polymorphic characters using default parameters for the character data sets.

## RESULTS

### *S*. Infantis demographics

A total of 15,706 *S*. Infantis WGS FASTA files available on NCBI as of 12 August 2024 and the 100 publicly available WGS FASTA files of *S*. Infantis from EnteroBase as of 3 December 2025 were downloaded. Source, country distributions, and collection years of all isolates are summarized in Table 1. The genome quality control metrics are provided in Table S7. Overall, most isolates originated from poultry and human sources, and the majority were from the United States. Most isolates were recovered in 2016–2023. In addition, SNP-typing data were also extracted from the NCBI isolates. Among the 14,415 isolates with reported SNP cluster results, the most common type was PDS000248108.48, which was detected in 7,757 (53.81%) of the isolates. Overall, there were 566 distinct SNP clusters identified among the isolates. Detailed metadata for each isolate are provided in Table S1.

**TABLE 1:** Isolate demographics

Isolate source	Number	Percent	Country isolated	Number	Percent	Year isolated	Number	Percent
Chicken	8,209	51.94	United States	10,655	67.41	2022	2,173	13.75
Human	3,919	24.79	United Kingdom	2,529	16.00	2021	1,990	12.59
Porcine	1,152	7.29	Canada	573	3.63	2018	1,809	11.45
Turkey	321	2.03	South Africa	373	2.36	2019	1,790	11.32
Food	314	1.99	Peru	238	1.51	2017	1,514	9.58
Bovine	292	1.85	Germany	226	1.43	2020	1,435	9.08
Environmental	256	1.62	Slovenia	161	1.02	2023	1,295	8.19
Animal	138	0.87	Other	1,051	6.65	2016	850	5.38
Avian	188	1.19				2015	604	3.82
Animal feed	77	0.49				2024	519	3.28
Egg	20	0.13				2014	396	2.51
Equine	15	0.09				2012	236	1.49
Canine	13	0.08				2013	215	1.36
Caprine	10	0.06				2010	196	1.24
Fish	7	0.04				2009	165	1.04
Camel	5	0.03				2011	160	1.01
Cat	2	0.01				2007	73	0.46
Mouse	1	0.01				2008	73	0.46
Shrimp	1	0.01				2000	46	0.29
Not reported	866	5.48				2006	30	0.19
						2004	27	0.17
						2005	25	0.16
						2001	17	0.11
						2003	17	0.11
						2002	14	0.09
						<2000	37	0.23
						Unknown	100	0.63

**Fig 1:**
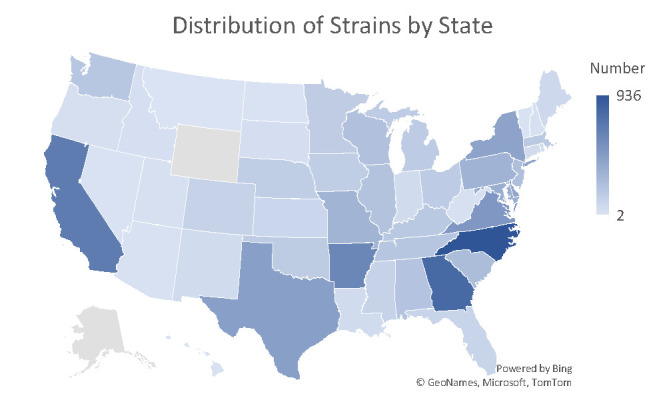
Geographic distribution of *S*. Infantis isolates across the United States. The heatmap on the right displays the number of isolates reported from each state.

For U.S. isolates, state-level and source distributions are shown in Table 2, with only states contributing more than 1% of isolates listed. The distribution of strains by state is visualized in Fig. 1.

**TABLE 2:** Isolate demographics

State isolated	Number	Percent	Isolate source	Number	Percent
NC	936	9.79	Chicken	7,783	73.05
GA	806	8.43	Swine	1,136	10.66
CA	679	7.10	Human	574	5.39
AR	608	6.36	Turkey	315	2.96
VA	501	5.24	Cattle	286	2.68
TX	449	4.70	Unknown	258	2.42
NY	422	4.41	Environmental	105	0.99
DE	403	4.22	Food	79	0.74
MD	352	3.68	Animal feed	43	0.40
PA	320	3.35	Animal	15	0.14
MO	313	3.27	Horses	14	0.13
SC	254	2.66	Dogs	13	0.12
NJ	240	2.51	Goats	9	0.08
WI	224	2.34	Egg	8	0.08
IL	215	2.25	Fish	7	0.07
AL	210	2.20	Avian	4	0.04
TN	192	2.01	Camel	3	0.03
WA	188	1.97	Cat	2	0.02
KY	176	1.84	Mouse	1	0.01
MA	172	1.80			
OK	160	1.67			
OH	159	1.66			
MI	157	1.64			
MN	150	1.57			
NE	149	1.56			
IA	148	1.55			
CO	116	1.21			
MS	112	1.17			
FL	103	1.08			

### Antimicrobial resistance

**Fig 2:**
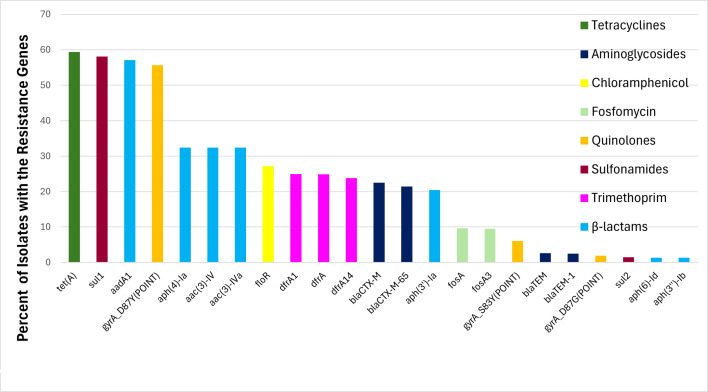
Percentage of *S*. Infantis isolates carrying the indicated AMR genes, with bar colors representing antimicrobial classes.

A total of 153 AMR genes were detected in at least one of these isolates, and 68.24% of the isolates (*N* = 10,786) carried at least one AMR gene. The most frequently detected resistance genes were *tet(A)* (tetracycline resistance; *N* = 9,385; 59.38%), *sul1* (sulfonamide resistance; *N* = 9,177; 58.06%), and *aadA1* (aminoglycoside resistance; *N* = 9,026; 57.10%). The AMR genes present in more than 1% of the isolates are shown in Fig. 2, and the AMR genes within each isolate are shown in Table S2. Notably, several genes were found in more than 20% of strains, including *tet(A)*, *sul1*, β-lactamase genes (*bla*_CTX-M_ and *bla*_CTX-M-65_), aminoglycoside resistance genes [*aadA1*, *aph(4)-la*, *aac(3)-IV*, *aac(3)-IVa*, and *aph(3′)-Ia*], trimethoprim resistance genes (*dfrA*, *drfA1*, and *dfrA14*), and the chloramphenicol resistance gene *floR*. Additionally, 55.68% of isolates had the *gyrA* D87T point mutation associated with resistance to quinolones, which are used to treat various infections, including non-typhoidal salmonellosis.

**Fig 3:**
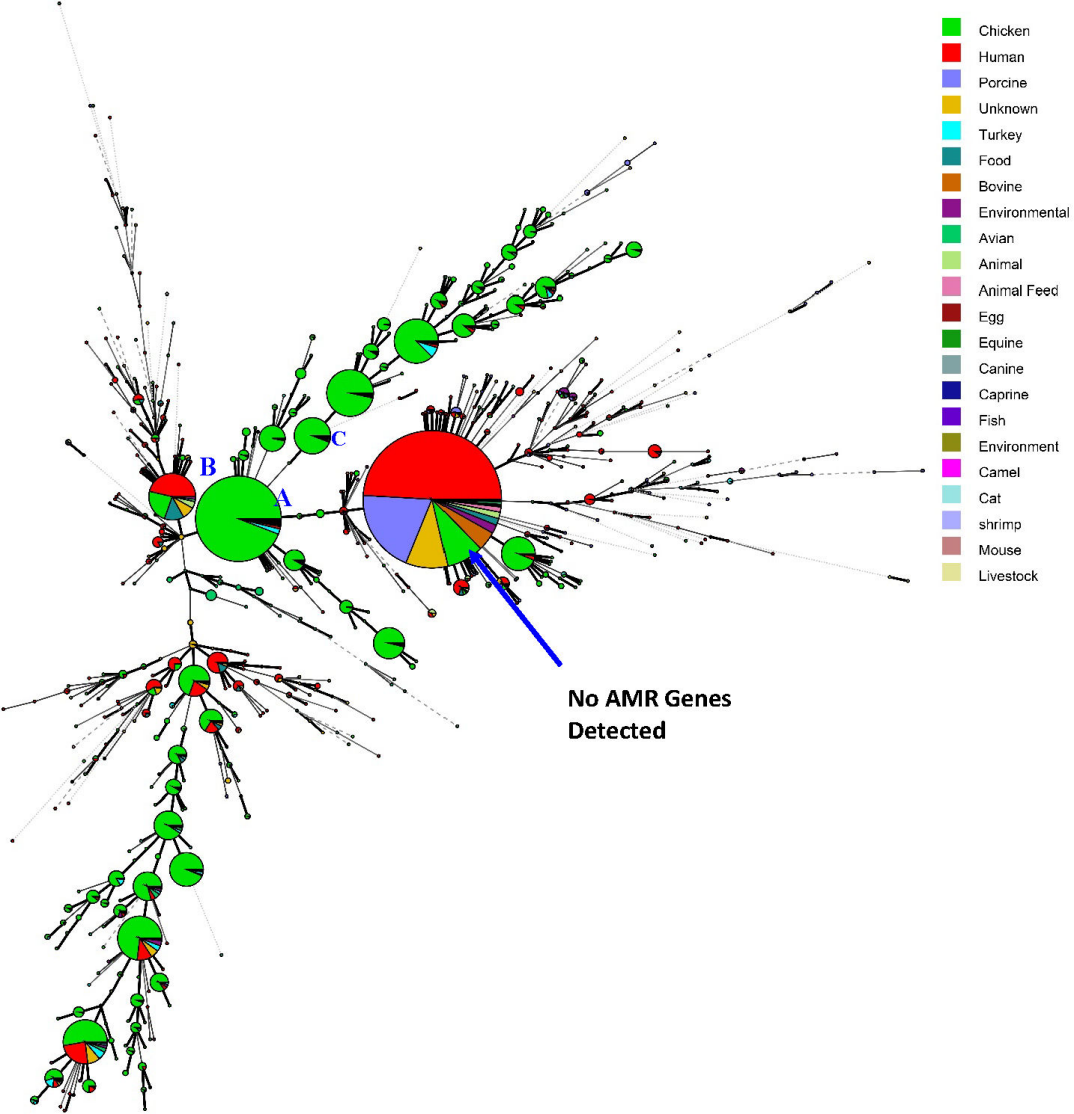
Minimum spanning tree analyses based on the AMR gene profiles of the *S*. Infantis strains included in the study. The trees are color coded based on isolation source. Each node represents an isolate, and node size is proportional to the number of isolates within that cluster. The larger circle in the bottom represents the strains without any identified AMR gene, and circles in the upper and middle represent the strains that contain at least one AMR gene. Major subgroups discussed in the main body are labeled with letters A to C in the figure.

Minimum spanning tree analysis separated the isolates into two main groups: one containing isolates with no detected AMR genes (the larger circle at the bottom in Fig. 3; Fig. S1) and the other containing isolates with at least one AMR gene, predominantly from chicken sources. The AMR-positive group was further divided into several subgroups. The specific differences among subgroups labeled A, B, and C in Fig. 3 are as follows: isolates in subgroup A contained *aadA1*, *sul1*, *tet(A)*, and *gyrA*_D87Y. In addition to the AMR genes present in subgroup A, isolates in subgroup C also harbored *aac(3)-IV*, *aac(3)-Iva*, and *aph(4)-Ia*, whereas isolates in subgroup B had only point mutation *gyrA*_S83Y. No significant difference in the AMR profiles was observed across isolation years (Fig. S1A). Figure S1B presents the distribution of isolates by geographic origin. AMR rates varied substantially among countries. Among countries with large sample sizes, the United States exhibited a high AMR rate (77.53%; 8,261/10,655), while the United Kingdom showed a moderate AMR prevalence (41.91%; 1,060/2,529). The majority of the isolates from the United States were identified as being SNP type PDS000248108.48 (67.36%; 7,177/10,655), which commonly carries multiple AMR genes (Table S3) and has been commonly detected in public health surveillance sampling of chickens (10). In contrast, Canada and South Africa demonstrated comparatively low AMR rates (18.15%, 104/573, and 12.33%, 46/373, respectively), with the majority of isolates lacking detectable AMR genes. Among countries with intermediate sample sizes, Australia displayed a near-even split between AMR-positive and AMR-negative isolates (52.94% AMR, 126/238), while Germany exhibited a high AMR prevalence (75.22%, 170/226). Several countries with smaller sample sizes, including Slovenia, Chile, Italy, Austria, and Ecuador, displayed very high AMR rates, ranging from 90.68% to 100%.

Pan-susceptible isolates (no detected AMR genes) accounted for 31.76% of the data set (*N* = 5,020). These isolates originate from diverse sources and span multiple countries and collection years. In terms of geographic distribution, a substantial majority of these isolates originate from North America and Europe. The top five countries (United States, United Kingdom, Canada, South Africa, and Australia) account for over 95% of all isolates analyzed. The proportion of pan-susceptible isolates varied significantly by country, ranging from <1% to 100%. High proportions were observed in Australia (88.19%), South Africa (87.67%), and Canada (81.85%), whereas the United States showed a low proportion (22.47%). The United Kingdom exhibited a moderate proportion of pan-susceptible isolates (58.09%). Several countries with very small sample sizes reported 100% pan-susceptibility, but these results are limited by low isolate numbers. Conversely, countries with larger sample sizes and very low proportions of pan-susceptible isolates included Peru (0.43%), Hungary (1.59%), Turkey (3.85%), Italy (6.20%), Chile (8.82%), and Slovenia (9.32%). Germany also exhibited a low proportion of pan-susceptible isolates (24.78%). Considerable variation in pan-susceptibility was observed across different sources. Human-sourced isolates accounted for the largest number of pan-susceptible isolates (*N* = 2,458), representing 62.72% of 3,919 total isolates from human patients. High proportions of pan-susceptible isolates were also observed among porcine (86.28%), bovine (76.03%), animal feed (77.92%), caprine (90.00%), and egg-derived isolates (100%), though caprine and egg sources had limited sample sizes. Environmental and food sources showed moderate proportions of pan-susceptible isolates at 43.75% (112/256) and 27.39% (86/314), respectively. Fish, equine, canine, and feline sources exhibited intermediate proportions of pan-susceptible isolates, ranging from 50.00% to 71.43%, though these categories were represented by relatively small sample sizes. In contrast, poultry-associated sources, particularly chicken and turkey, showed markedly low proportions of pan-susceptible isolates. Only 5.34% of chicken isolates (438/8,209) and 5.61% of turkey isolates (18/321) lacked detectable AMR genes. Avian sources overall also showed low pan-susceptibility (9.04%). This observed high level of AMR genes coincides with a high proportion of chicken (83.03%; 6,816/8,209) and turkey (85.67%, 275/321) isolates being of SNP type PDS000248108.48, where at least 87% of isolates carry *tet(A)*, *sul1*, and *aadA1* (Table S3). Temporal analysis revealed substantial variation across years. Overall, earlier years showed higher proportions of pan-susceptible isolates. Isolates collected prior to 2000 exhibited the highest proportion of pan-susceptible isolates (83.78%, 31/37). Similarly high percentages were observed during the early 2000s, with values exceeding 70% in most years between 2002 and 2009, including peaks in 2006 (76.67%), 2009 (76.36%), and 2003 (76.47%). These years, however, were generally represented by relatively small total isolate numbers. Between 2010 and 2016, the proportion of no AMR isolates remained moderate, ranging from 36.88% in 2011 to 57.88% in 2016. Notably, 2015 and 2016 showed over half of isolates classified as no AMR (54.80% and 57.88%, respectively), despite increasing sample sizes compared with earlier years. From 2017 onward, a pronounced downward trend was observed. While 2017 and 2021 showed transient increases in pan-susceptible isolates (47.29% and 46.23%, respectively), the majority of recent years demonstrated substantially lower values. In particular, 2018–2020 showed a decline from 24.10% to 20.17%, followed by a further reduction in 2022 (10.03%) and 2023 (10.58%). The lowest proportion of isolates with no AMR genes reported was observed in 2024, with only 5.78% (30/519) classified as not carrying an AMR gene. Detailed frequencies of all AMR identified are listed in Table S2.

### Plasmid characterization

Plasmid conjugation is a key mechanism through which bacteria acquire new genes, leading to antimicrobial resistance and the emergence of novel phenotypes. Identifying transfer-related genes associated with different plasmid types is a valuable approach for predicting the plasmid types present and recognizing potential limitations to the conjugative ability when these genes are absent. To predict the plasmids present in these isolates, the transfer genes associated with various plasmid types were analyzed using the described Plasmid Transfer Gene database (19). Among the 12 major plasmid types examined, each was detected in some isolates, with all containing at least one associated transfer gene (Table S4). Of these plasmid types, IncI1-associated transfer genes were the most prevalent, with 66.27% (*N* = 10,474) of the isolates containing at least one associated gene (Table 3; Table S4). Notably, 180 of these isolates possessed all 42 IncI-associated transfer genes; 28 isolates had 41; and 9,055 isolates had 40 of these transfer-associated genes (Table S3). Additionally, 800, 157, 91, and 163 isolates contained 30–39, 20–29, 10–19, and 1–9 IncI1-associated transfer genes, respectively (Table S4). The IncI1 plasmids were widely distributed across the different SNP types, with all but 3 of the top 60 most detected SNP types having at least one isolate carrying at least five of the IncI1 transfer genes (Table S3). Notably, 98.66% (7,653/7,757) of the PDS000248108.48 isolates carried the IncI1-plasmid replicons, which likely corresponded to the pESI plasmid. IncB/O/K was the second most prevalent plasmid type, found in 33.11% of the isolates (*N* = 5,233), each carrying at least one associated transfer genes (Table S4). Among these isolates, seven contained all 38 IncB/O/K-associated transfer genes. The numbers of isolates containing 29, 27, 5, 4, 3, and 2 transfer genes associated with IncB/O/K plasmids were 2, 1, 1, 13, 10, and 7, respectively; all of these isolates contained the IncB/O/K*_pilV* gene. Among the remaining 5,192 isolates that contained only one IncB/O/K-associated transfer gene, 5,080 harbored the IncB/O/K_*pilV* gene, which is likely a false positive due to sequence overlap with the IncI1_*pilV* gene (19). Transfer genes associated with IncA/C (*N* = 384, 2.43%) and IncN (*N* = 337, 2.13%) plasmids were detected in more than 2% of the strains, while the remaining eight plasmid types were detected in less than 1% of the isolates. Detailed results for plasmid transfer genes are presented in Table S4 in the supplemental data.

**TABLE 3:** Plasmid transfer genes detected

Plasmids	Number of isolates^*a*^	Percent
IncI1	10,474	66.27
IncB/O/K	5,233	33.11
IncAC	384	2.43
IncN	337	2.13
IncFIA	155	0.98
IncFIB	140	0.89
IncHI2	96	0.61
IncHI1	49	0.31
IncI2	31	0.2
IncM	17	0.11
IncP	13	0.08
IncW	3	0.02

^*a*^ Number of isolates containing at least one of the genes.

The pESI plasmids carry multiple AMR and virulence genes, which are important for understanding the pathogenic potential of this bacterium. pESI plasmids may play a significant role in the global emergence of *S*. Infantis populations. To compare the results of the plasmids detected in this study with prototypical pESI plasmids, six defined pESI plasmids were randomly selected, and their sequences were downloaded from NCBI and analyzed using the Plasmid Transfer Gene database to determine their transfer gene profiles (19). The results show that all six pESI plasmids carried a cohort of multiple IncI1 plasmid-associated genes, the most prevalent plasmid type identified in the study. Among the 42 transfer genes associated with IncI1 plasmids, 40 were present in these pESI plasmids, with exceptions being *pilJ* and *traD*. Interestingly, among the 9,055 isolates in this study that contained 40 IncI1-associated genes, only 1 isolate (GCA_009150245.1) showed the exact same transfer gene profile as the six pESI reference plasmids (Table S4). The remaining 9,054 isolates lacked *pilI*, rather than *pilJ*. In these isolates, 52.88% (*N* = 4,789) also contained IncB/O/K*_pilV*, which, due to sequence overlap with IncI1_*pilV*, may represent cross-reactivity rather than a true positive for the IncB/O/K plasmid replicon. Overall, the gene IncI1*_pilI* was detected in only 1.34% (*N* = 212) of the isolates, whereas IncI1*_pilJ* was present in 61.24% (*N* = 9,679) of the isolates.

Previous studies have shown that in addition to a critical role in the spread of AMR, pESI plasmids also contribute to *Salmonella* virulence (13). When the WGS of the pESI plasmids was assessed using the *Salmonella* Virulence Factor Comparison tool, all six plasmids contained only one virulence gene (*STY_RS12400-STY2631*), located on the novel *Salmonella* Pathogenicity Island SPI-17. When assessed using the *E. coli* Virulence Factor Comparison tool, four more virulence genes (*ybtQ*, *fyuA*, *irp12*, and yfeA) were detected in all the six plasmids. Two additional virulence genes (*yfeB* and *cib*) were detected in plasmid pESI-CTX-M-65.

**Fig 4:**
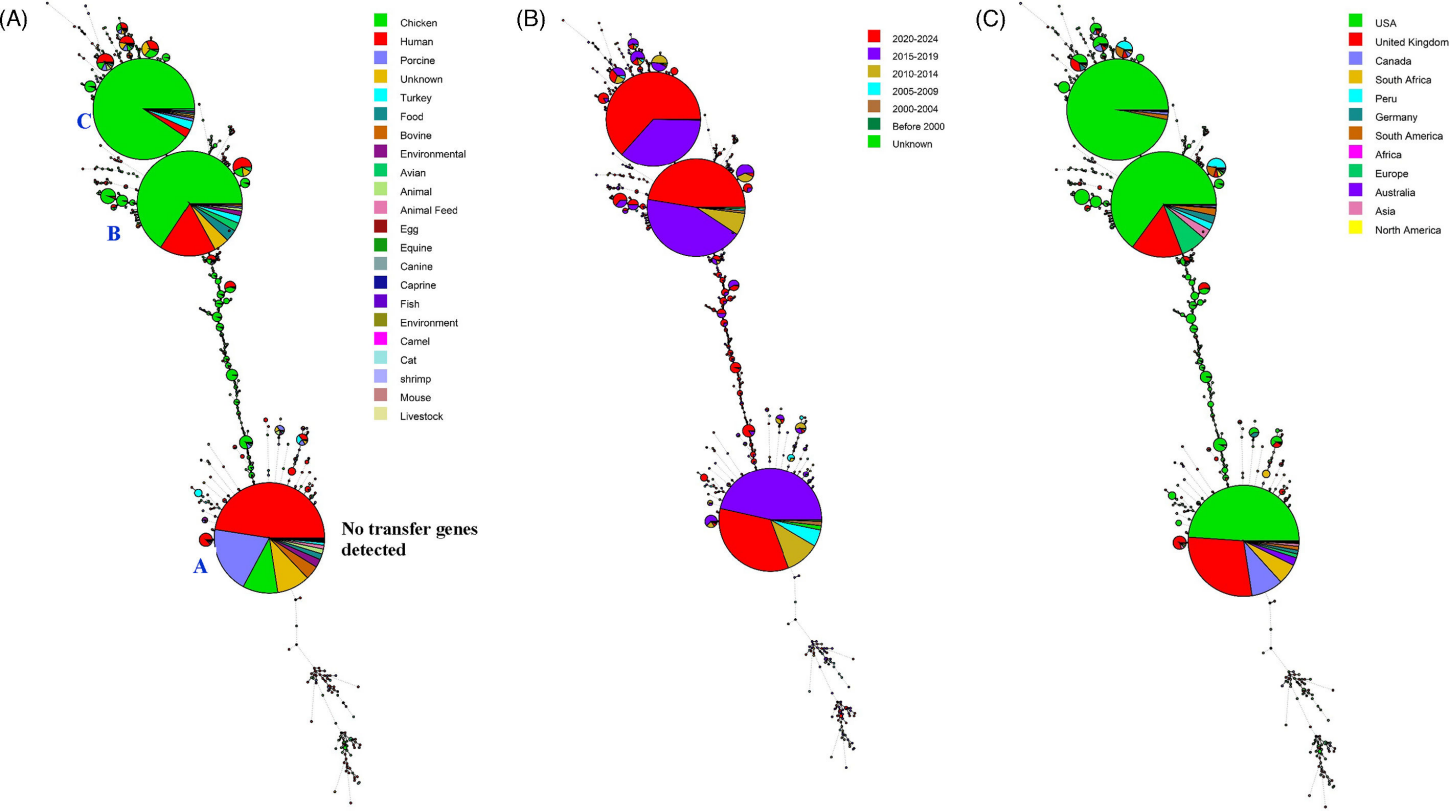
Minimum spanning tree analyses based on the plasmid transfer genes. The trees are color coded based on isolation source (**A**), 5-year isolation period (**B**), or country of origin (**C**). In panel C, the six countries with more than 200 isolates are shown individually, while isolates from all remaining countries are grouped by continent, excluding those already represented among the most common countries. The relative size of the circles is proportional to the group size. Major subgroups discussed in the main body are labeled with letters A to C in the figure.

When the profiles of the transfer genes were analyzed using minimum spanning tree analysis, the isolates were clustered into three major groups (Fig. 4). The isolates in group A lacked all the transfer genes examined in this study, and nearly half of the isolates were from human patients. The isolates in group B and C contained all the IncI1-associated transfer genes except *pilJ* and *traD*. The majority of isolates in group C were from chickens, while group B included isolates from both chicken and human sources (Fig. 4).

### Virulence gene characterization

To identify the VFs present in the isolates, the WGS data for each strain were analyzed using the *Salmonella* Virulence Factor Comparison tool (18). Of the 594 genes examined, 152 (25.8%) were present in all isolates. The remaining genes were distributed as follows: 279 (47.3%) were found in more than 99% of the strains, 3 in 98%–99%, 1 in 96.5%, and 145 (24.6%) in between 0% and 1.6% of isolates. Fourteen (2.4%) of the genes were absent in all isolates (Table S5). Genes consistently present in all or more than 99% of the isolates included those encoding fimbriae (e.g., *bcd*, *csg*, *fim*, *stf*, *std*, and *tcf* operons), flagellin (*fliC*), two-component regulators (*cpxA*/*cpxR* and *phoP*/*phoQ*), and type III secretion systems (e.g., *ssa* and *sse* operons) (Table S5). The *tviABCDE* and *vexABCDE* genes, encoded in SPI-7 that is most commonly associated with *S*. Typhi (23), were absent in all strains (Table S5).

**Fig 5:**
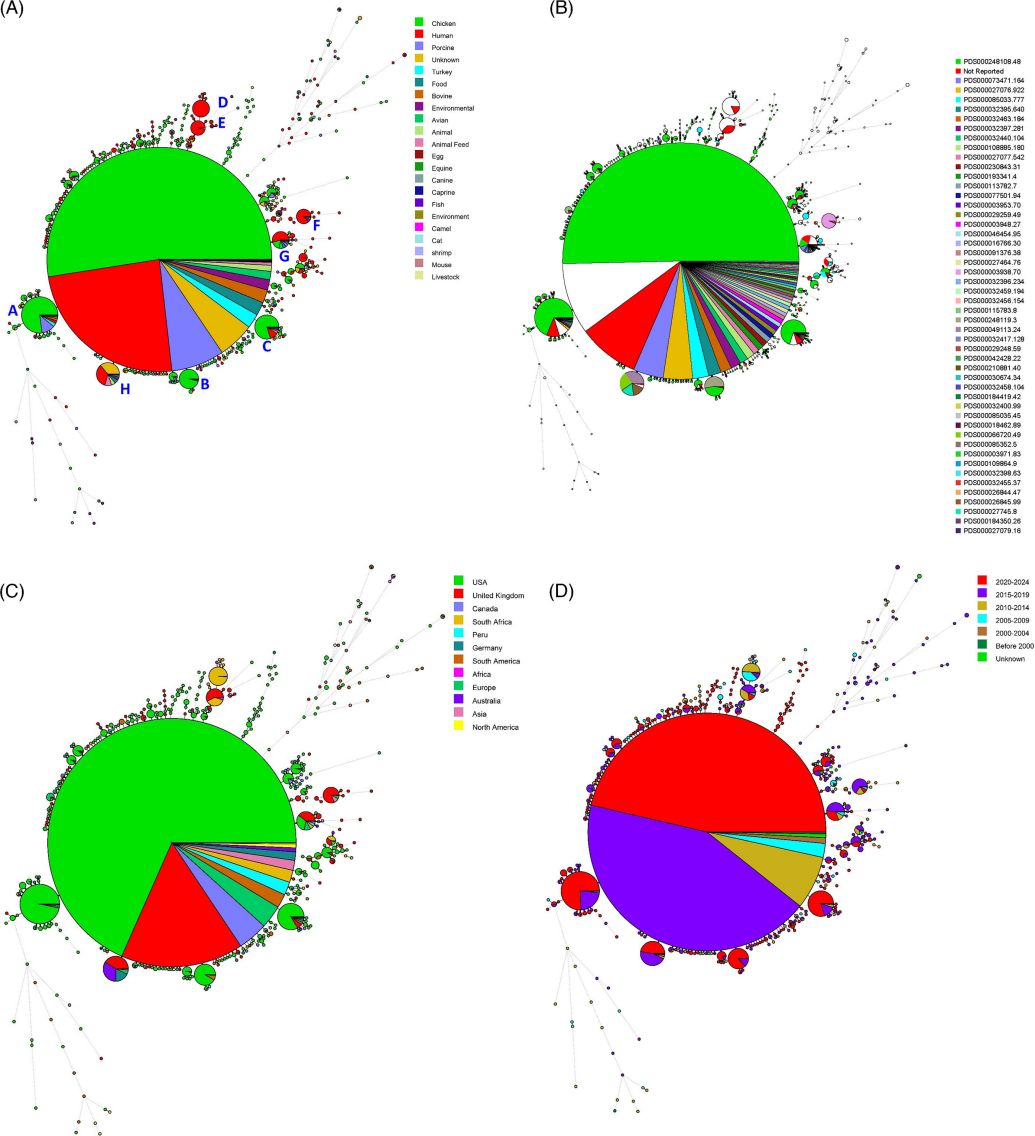
Minimum spanning tree analyses based on the phylogenetically relevant virulence genes of the *S*. Infantis strains included in the study. The trees are color coded based on isolation source (**A**), SNP type (**B**), country of origin (**C**), or year (**D**). The six countries with more than 200 isolates are shown individually, while isolates from all remaining countries are grouped by continent, excluding those already represented among the most common countries. For the SNP-typing results, the top 50 SNP-types are displayed. The relative size of the circles is proportional to the group size. Major subgroups discussed in the main body are labeled with letters A to H in the figure.

The VFs were further analyzed using phylogenetic analyses, and minimal spanning trees were constructed to explore the diversity by isolation sources, geographic location, and year of isolation. As shown in Fig. 5, the majority of the isolates (87.45%; *N* = 13,822) clustered into a single large group, suggesting a high degree of clonal relatedness despite diverse sources. This group shared 435 of the 594 putative virulence genes screened. The background genetic diversity of this group was also quite diverse, with a wide diversity of SNP types detected at approximately the same proportions as the overall *S*. Infantis data set. Eight additional groups contained more than 60 strains per group and were labeled from A to H (Fig. 5). The majority of isolates from groups A to C originated from chicken-related sources, whereas groups D–H were predominantly from human patients (Fig. 5A). The majority of groups A–C strains were SNP type PDS000248108.48, which was by far the most common SNP type detected among the strains (Fig. 5B). Group D and E strains either did not have a SNP type reported or were made of less prevalent SNP types (i.e., other SNP types outside of the top 30). Groups F, G, and H contained isolates from multiple sources, indicating that the virulence gene profiles of human isolates exhibit diverse yet overlapped profiles from food and animal-related sources. The predominant SNP type for group F was PDS000003938.70; for group H, it was PDS00049113.24, PDS000066720.49, and PDS000027745.8, while group G displayed significant diversity of SNP types (Fig. 5B). Differences in VF profiles among groups were limited. Relative to the main cluster, groups A lacked *tinR*; group B lacked *nmpC*; and group C lacked *wcaM* and *galF*. Compared to the isolates in the largest group, the isolates in group D carried *sopE*, *int*, and *xis* while lacking the *sciL*, *sciR*, and *sciS*. Group E isolates possessed *int* and *xis* but lacked the gene *STM0438*. Group F isolates possessed putative virulence genes S004 and S013–S026, which were absent from the largest group. Group G isolates uniquely carried *traT*, detected in 0.8% (*N* = 129) of all isolates. Detailed VF profiles for each group are provided in Table S6.

When examining the VF profiles based on geographic location, the majority of strains in groups A and B are from the United States, while most strains in group D originated from South Africa. The isolates in the other four groups were derived from a broader geographic range (Fig. 5C).

The comparison of these VFs across different isolation sources is shown in Table 4. Genes such as *STM0438*, *sciL*, *sciR*, *sciS*, *nmpC*, and *tinR* were consistently detected across all source categories, with some showing 100% detection in specific sources. Conversely, other genes are found in low quantities across all isolate sources. For example, *Salmonella* genomic island 1 (SGI-1)-associated genes, *S004* and *S013-S026*, were absent in the isolates from the generic animal, animal feed, avian, dogs, goats, cat, chicken, egg, mouse, swine, shrimp, or turkey.

**TABLE 4:** Percentage of strains with the respective virulence gene by source type

Source	Animal (*N* = 137)	Animal feed (*N* = 77)	Avian (*N* = 98)	Bovine (*N* = 292)	Camel (*N* = 5)	Canine (*N* = 13)	Caprine (*N* = 10)	Cat (*N* = 2)	Chicken (*N* = 8,209)	Egg (*N* = 20)	Environmental (*N* = 62)	Equine (*N* = 15)	Fish (*N* = 7)	Food (*N* = 314)	Human (*N* = 3,917)	Mouse (*N* = 1)	Porcine (*N* = 152)	Shrimp (*N* = 1)	Turkey (*N* = 321)	Unknown (*N* = 3,866)
*STM0438*	98.54	97.4	98.94	100	100	92.31	100	100	99.91	100	96.39	93.33	100	98.09	95.56	100	99.83	100	99.69	99.54
*sciL*	100	97.4	100	100	100	100	100	100	99.91	100	97.19	100	100	99.04	97.27	100	99.91	100	99.69	99.77
*sciR*	99.27	100	100	100	100	100	100	100	99.93	100	97.59	100	100	99.68	97.29	100	99.74	100	100	100
*sciS*	100	100	100	100	100	100	100	100	99.96	100	97.59	100	100	99.68	97.32	100	99.91	100	99.69	100
*tinR*	97.08	97.4	96.94	95.89	100	100	100	100	95.46	90	96.79	100	100	97.45	98.83	100	94.88	100	94.7	97.81
*nmpC*	100	100	100	99.66	100	100	100	100	98.65	100	100	100	100	99.68	99.82	100	100	100	100	100
*sopE*	1.46	0	0	0	0	0	0	0	0.07	0	1.61	0	0	1.27	4.72	0	0.09	0	0.31	0.35
*int*	2.19	1.3	0	1.03	0	0	0	0	0.17	5	3.21	6.67	0	1.27	5.08	0	0.26	0	0.31	0.81
*xis*	2.19	1.3	0	1.03	0	0	0	0	0.17	5	2.41	6.67	0	1.27	4.93	0	0.26	0	0.31	0.81
*STY_RS23105-*	6.57	22.08	1.02	0.34	0	0	0	0	0.06	0	3.61	6.67	0	2.87	1.35	0	0.09	0	0	7.04
*S004*	0	0	0	0	0	0	0	0	0	0	1.61	0	14.29	0.32	1.84	0	0	0	0	0.35
*S013*	0	0	0	0	0	0	0	0	0	0	1.61	0	14.29	0.32	1.84	0	0	0	0	0.35
*S014*	0	0	0	0	0	0	0	0	0	0	1.61	0	14.29	0.32	1.84	0	0	0	0	0.35
*S015*	0	0	0	0	0	0	0	0	0	0	1.61	0	14.29	0.32	1.66	0	0	0	0	0.23
*S016*	0	0	0	0	0	0	0	0	0	0	1.61	0	14.29	0.32	1.66	0	0	0	0	0.23
*S017*	0	0	0	0	0	0	0	0	0	0	1.61	0	14.29	0.32	1.66	0	0	0	0	0.23
*S018*	0	0	0	0	0	0	0	0	0	0	1.61	0	14.29	0.32	1.66	0	0	0	0	0.23
*S019*	0	0	0	0	0	0	0	0	0	0	1.61	0	14.29	0.32	1.66	0	0	0	0	0.23
*S020*	0	0	1.37	20	0	0	0	0	0	0	3.21	13.33	14.29	0.64	1.94	0	0	0	0	0.46
*S021*	0	0	1.03	20	0	0	0	0	0	0	3.21	13.33	14.29	0.64	1.94	0	0	0	0	0.46
*S022*	0	0	1.03	20	0	0	0	0	0	0	3.21	13.33	14.29	0.64	1.94	0	0	0	0	0.46
*S023*	0	0	0	0	0	0	0	0	0	0	1.61	0	14.29	0.32	1.56	0	0	0	0	0.23
*S024*	0	0	0	0	0	0	0	0	0	0	1.61	0	14.29	0.64	1.53	0	0	0	0	0.23
*S025*	0	0	1.03	20	0	0	0	0	0	0	3.21	13.33	14.29	0.64	1.61	0	0	0	0	0.35
*S026*	0	0	1.03	20	0	0	0	0	0	0	3.21	13.33	14.29	0.32	1.61	0	0	0	0	0.35

## DISCUSSION

As one of the most frequently reported non-typhoidal *Salmonella* serovars worldwide, *S*. Infantis has disseminated globally across humans, animals, food, and environmental reservoirs, with a steadily increasing burden over recent decades. Notably, multidrug-resistant S. Infantis has emerged and spread worldwide (1). The large-scale genomic analysis of the AMR, virulence, and plasmid transfer-associated genes can enhance understanding of resistance dissemination and pathogenicity in *S*. Infantis while informing surveillance efforts and guiding targeted interventions to mitigate the spread of multidrug-resistant *S*. Infantis.

The AMR gene analysis reveals a substantial global burden of AMR among *S*. Infantis isolates analyzed in this study, with more than two-thirds of isolates carrying at least one AMR gene. Importantly, substantial geographic heterogeneity in AMR prevalence was observed, and these patterns persist even after normalizing for sampling size by examining resistance rates rather than raw isolate counts. This indicates that differences in AMR prevalence cannot be explained solely by uneven data contributions to public databases. High-income countries with extensive surveillance programs, particularly the United States and Germany, exhibited high AMR rates and low proportions of pan-susceptible isolates. The United States contributed the largest number of isolates (*N* = 10,655), yet only 22.47% lacked AMR genes, reflecting a substantial resistance burden. An underlying reason for this observation is that a relatively high proportion of the isolates from the United States originated from chickens (73.05%; *N*=7,783/10,655), and globally, most of the chicken isolates (85.13%; *N*=6,916/8,007) belonged to SNP type PDS000248108.48, which has a high preponderance of AMR. Germany showed a similarly low proportion of isolates without any AMR genes (24.78%). These findings may reflect greater antimicrobial exposure in food animal production, intensive surveillance of high-risk reservoirs, and more comprehensive detection of resistance determinants. This finding is consistent with a recent study reporting a high prevalence of MDR *S*. Infantis isolates in the U.S. and European WHO regions (1). In contrast, Australia (88.19%), South Africa (87.67%), and Canada (81.85%) demonstrated high proportions of pan-susceptible isolates, suggesting comparatively lower AMR prevalence in these data sets. These countries were dominated by isolates of SNP types PDS000027076.922 (Canada), PDS000113782.7 and PDS000030674.34 (South Africa), and PDS000049113.24 (Australia), which were types that had a low prevalence of plasmids and subsequently AMR genes (Table S3). These patterns may be influenced by differences in antimicrobial usage policies, dominant reservoirs, clonal expansion of key strains, agricultural practices, or broader inclusion of susceptible isolates through routine surveillance programs. Several countries reported extremely high percentages (100%) of pan-susceptible isolates, including Nigeria, Cameroon, Denmark, Kenya, and Senegal; however, these observations were based on very small sample sizes (*n* ≤ 3) and should therefore be interpreted with caution. Similarly, extremely high AMR rates observed in countries with limited sample sizes, such as Austria, Ecuador, Italy, and Slovenia, likely reflect targeted sequencing or reporting biases, where resistant isolates are preferentially submitted, rather than true population-level prevalence. These findings underscore the importance of considering surveillance intensity and sampling design when interpreting global AMR patterns. Nonetheless, several countries with moderate to large sample sizes exhibited alarmingly low proportions of pan-susceptible isolates, including Peru (0.43% of 238 isolates), Hungary (1.6% of 63 isolates), Turkey (3.9% of 52 isolates), Italy (6.2% of 129 isolates), Chile (8.8% of 136 isolates), and Slovenia (9.3% of 161 isolates). Isolates from these countries were predominated by SNP types that commonly carry the IncI1 plasmid genes and corresponding AMR genotypes (Table S3). These results suggest a high prevalence of resistance in these regions and raise concerns regarding treatment effectiveness and public health risk. The predominance of resistance genes such as *tet(A)*, *sul1*, and *aadA1* is consistent with widespread exposure to tetracyclines, sulfonamides, and aminoglycosides in food animal production systems (24). These resistance genes were very common among the SNP type PDS000248108.48 strains. For example, 89.36%, 88.02%, and 87.75% of the 7,757 SNP type PDS000248108.48 strains carried *tet(A)*, *sul1*, and *aadA1*, respectively. The frequent detection of clinically important β-lactamase genes compromises recommended treatment options for the management of salmonellosis (25). β-Lactamase genes, such as *bla*_CTX-M_ and *bla*_CTX-M-65_, were detected in over 40% of the SNP type PDS000248108.48 strains, which is indicative of their challenges in chickens. Another particular concern is the high prevalence of *gyrA* point mutations associated with quinolone resistance, given the importance of fluoroquinolones in the treatment of invasive salmonellosis. The co-existence of chromosomal mutations and multiple acquired AMR genes highlights strong selective pressure and the adaptive capacity of *S*. Infantis. Compared with other isolation sources, poultry-associated isolates (chicken, turkey, and other avian sources) exhibited significantly higher rates of AMR-positive profiles. This observation is consistent with a previous study identifying poultry and chicken meat as primary sources of multidrug-resistant *S*. Infantis (1) and supports their role as major reservoirs for AMR *S*. Infantis. Temporal analysis demonstrated a clear decline in pan-susceptible isolates over time, particularly after 2017, indicating a progressive increase in AMR prevalence. This trend likely reflects cumulative antimicrobial exposure and the successful dissemination of resistant clones. Although year-to-year fluctuations were observed, the overall trajectory points toward increasing resistance in recent years, posing a growing public health challenge.

This comprehensive analysis of plasmid transfer-associated genes highlights the dominant role of IncI1-like plasmids in the global *S*. Infantis population. The high prevalence of IncI1-associated transfer genes, detected in over two-thirds of the isolates, underscores the importance of IncI1 plasmids in mediating horizontal gene transfer and facilitating the dissemination of antimicrobial resistance in these isolates. The similarity in transfer gene profiles of the IncI1 plasmids and those of reference pESI plasmids supports previous findings that pESI plasmids contain a region originating from IncI1 plasmids (13, 26). The absence or variability of *pilI* and *pilJ* among most isolates suggests structural diversification of these plasmids, potentially reflecting adaptation to different hosts or ecological niches. Given that both *pilI* and *pilJ* are involved in type IV pilus assembly and conjugation, variation in these genes may influence plasmid transfer efficiency and dissemination dynamics. The frequent detection of IncB/O/K_*pilV*, particularly among isolates already carrying IncI1-associated genes, likely reflects sequence overlap rather than true co-carriage, emphasizing the need for cautious interpretation of single-gene plasmid predictions. The presence of complete IncB/O/K transfer gene sets, particularly among isolates already carrying IncI1-associated genes, indicates that additional plasmid types may contribute to genetic exchange in a subset of isolates. The identification of virulence-associated genes on pESI plasmids reinforces their role in enhancing the pathogenic potential of *S*. Infantis. The presence of yersiniabactin-associated genes is consistent with previous studies showing that pESI harbors the yersiniabactin siderophore cluster (*ybt*, *fyuA*, and *irp12*) involved in iron acquisition during infection (13). The additional virulence genes *yfeA* and *yfeB* encode proteins that are part of a bacterial metal ion transport system, which can play a crucial role in virulence, particularly under metal-limiting conditions such as those encountered during host infection (27). The *cib* gene is a bacteriocin-associated virulence factor that encodes colicin Ib, a protein toxin that targets and kills closely related bacterial strains, thereby reducing microbial competition (28). These additional virulence factors detected in specific plasmid variants further suggest plasmid-mediated enhancement of competitiveness and survival of the bacteria.

Virulence gene analysis revealed that the high prevalence of core virulence genes across isolates, regardless of isolation source or geographic origin, indicates a conserved virulence backbone in *S*. Infantis. The widespread presence of genes encoding fimbriae, flagellar components, two-component regulatory systems, and type III secretion systems underscores their essential roles in host colonization, survival, and pathogenicity. The complete absence of SPI-7-associated genes (*tviABCDE* and *vexABCDE*), which are characteristic of *S*. Typhi, is consistent with the non-typhoidal nature of the isolates analyzed. Phylogenetic clustering based on VF profiles revealed a high degree of clonal relatedness, as evidenced by the dominance of a single large cluster. Although isolates originated from diverse sources, VF differences among groups were minimal, suggesting that variation in virulence gene content is driven by a limited number of accessory elements rather than broad divergence in pathogenic potential. Several group-specific VF differences are biologically notable. The *tinR* gene encodes a transcriptional regulator involved in iron acquisition and regulation, particularly under iron-limited conditions. The *nmpC* gene encodes a major outer membrane protein that functions as a porin, modulating membrane permeability and contributing to antibiotic resistance (29). In *Salmonella*, the *wcaM* gene, part of the *wca* operon, is involved in capsular polysaccharide (colonic acid) synthesis, a key component of the bacterial capsule that helps *Salmonella* evade the immune system, survive in harsh conditions, and contribute to its virulence (30). The *galF* gene in *Salmonella* participates in the biosynthesis of the galactose-containing component of the lipopolysaccharide, a key structure in the outer membrane of gram-negative bacteria that supports resistance to host immune defenses and helps protect the bacterial cell from hostile environments (31). The absence of these genes in select chicken-associated groups may influence iron acquisition, membrane permeability, capsule formation, and lipopolysaccharide biosynthesis, potentially affecting host adaptation and environmental survival. In contrast, human-associated groups were enriched for phage- and genomic island-associated genes, including *sopE*, *int*, and *xis*. Notably, 81% (199/245) of *int*-positive and 81% of *xis*-positive (193/237) isolates were derived from human patients. Genes *int* and *xis* encode an integrase and an excisionase, respectively, which are involved in the integration and excision of bacteriophage DNA during the lytic cycle (32). These genes are located on SGI-1, which typically carries AMR genes (particularly those associated with MDR strains) and potential virulence determinants (33, 34). The predominance of *int* and *xis* among human isolates suggests a role for SGI-1 in human infection, particularly given its association with antimicrobial resistance and virulence determinants. The *sopE* is also phage-derived and encodes an effector of the SPI-1 type III secretion system, which facilitates host cell invasion by inducing membrane ruffling (23, 35, 36). The presence of sopE further supports enhanced invasion potential in certain human-associated isolates. Genes *sciL*, *sciR*, and *sciS* are core genes of the type VI secretion system, located in SPI-6, and play a critical role in bacterial virulence, host interactions, and competition with other microbes (23, 37). The absence of these genes in some groups may reflect differences in interbacterial competition or host interactions. Group F isolates were notable for carrying unique SGI-1-associated hypothetical virulence genes (*S004* and *S013–S026*), the majority of which were derived from human clinical samples, suggesting a possible contribution of these SGI-1-associated genes to human-specific pathogenicity. The *traT* gene, carried by isolates from group G, is the surface exclusion gene associated with IncF plasmids, which plays a crucial role in the immune evasion strategies of *Salmonella* by preventing complement-mediated killing and reducing phagocytosis (38).

When the VF, AMR, and plasmid transfer gene data sets for the strains were combined in a composite analysis, there were overlaps of multiple food animal sources, including porcine, chicken, and bovine sources, and those from human patients (Fig. S2A). Among the isolates of predominant SNP cluster PDS000248108.48 (Fig. S2B), there is some diversity across the composite data set, yet in some lineages, there is not much overlap with isolates from human patients, while on other composite lineages, there is more overlap. These observations indicate that there are some genes (such as specific AMR genes) that are more common in those PDS000248108.48 strains more associated with human infections. Overall, the study showed that VF prevalence differences across sources were minimal. However, there was some diversity in virulence profiles observed among isolates from human patients, suggesting that multiple VFs may contribute to the enhanced pathogenic potential of *S*. Infantis and its ability to survive in different host environments, evade immune responses, or cause more severe disease. The analysis of the plasmid transfer-associated genes highlights the central role of IncI1-like and pESI-related plasmids in shaping the AMR and virulence landscape of *S*. Infantis. Continued genomic surveillance and detailed characterization are essential to elucidate the roles and interactions of these genetic elements in pathogenesis.

### Conclusion

*S*. Infantis has been emerging as a significant public health concern due to its virulence and AMR profiles, posing threats to both human health and animal health. This study provides a comprehensive genetic analysis of a large number of isolates, identifying AMR genes, plasmid transfer-associated genes, and virulence factors across a wide range of isolates. The high AMR resistance rate, particularly within poultry-associated populations, highlights the global expansion of antimicrobial resistance in *S*. Infantis. Overall, the majority of the isolates shared similar VF profiles, with limited variation in VF prevalence. This may reflect an overrepresentation of pESI plasmid-carrying strains in GenBank due to heightened recent research interest and targeted surveillance efforts (9, 13, 39–41). Nevertheless, the observation that strains from human patients shared common VFs, plasmids, and AMR profiles with those from a wide range of different sources supports the potential of interenvironmental transmission, particularly via the food chain. These factors likely enhance the ability of *S*. Infantis to survive in host environments, evade immune responses, and potentially cause more severe disease. Furthermore, the findings lay a strong foundation for future investigations into the mechanisms driving the pathogenicity, spread, and outbreak potential of this emerging serotype.

## Data Availability

All data used in this study were obtained from the National Center for Biotechnology Information (NCBI) database and are publicly available.

## SUPPLEMENTAL MATERIAL

Figure S1A aem.01975-25-s0001.tiff Minimum spanning tree analyses based on the AMR profiles of the isolates, color coded based on years.

Figure S2A aem.01975-25-s0002.tiff Minimum spanning tree using a composite data set of polymorphic characters for the VFs, AMR genes, and plasmid transfer genes, color coded by source.

Figure S1B aem.01975-25-s0003.tiff Minimum spanning tree analyses based on the AMR profiles of the isolates, color coded based on locations.

Figure S2B aem.01975-25-s0004.tiff Minimum spanning tree using a composite data set of polymorphic characters for the VFs, AMR genes, and plasmid transfer genes, color coded by SNP type.

Supplemental legends aem.01975-25-s0005.docx Descriptive legends for Tables S1 to S7 and Fig. S1 and S2.

ASM does not own the copyrights to Supplemental Material that may be linked to, or accessed through, an article. The authors have granted ASM a non-exclusive, world-wide license to publish the Supplemental Material files. Please contact the corresponding author directly for reuse.
