# Supplementary figures and images for: Genomic characterization of antimicrobial resistance and virulence determinants in Salmonella Infantis isolated from human, food, and animal sources
Source: Appl Environ Microbiol. 2026 Mar 23;92(4):e01975-25. doi: 10.1128/aem.01975-25 (PMC13101500; doi:10.1128/aem.01975-25)

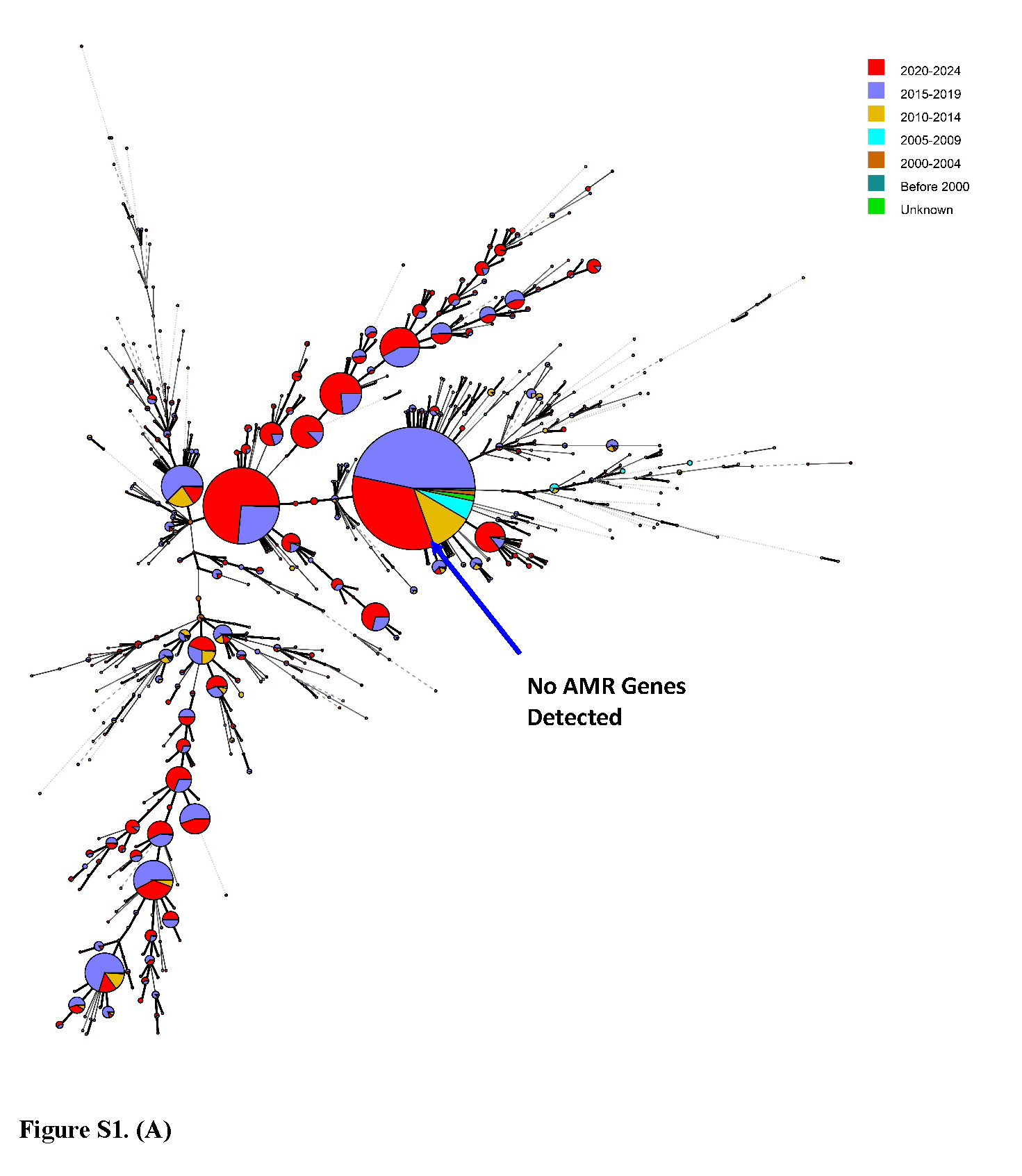

Supplement: Figure S1A — Minimum spanning tree analyses based on the AMR profiles of the isolates, color coded based on years. [file aem.01975-25-s0001.tiff]

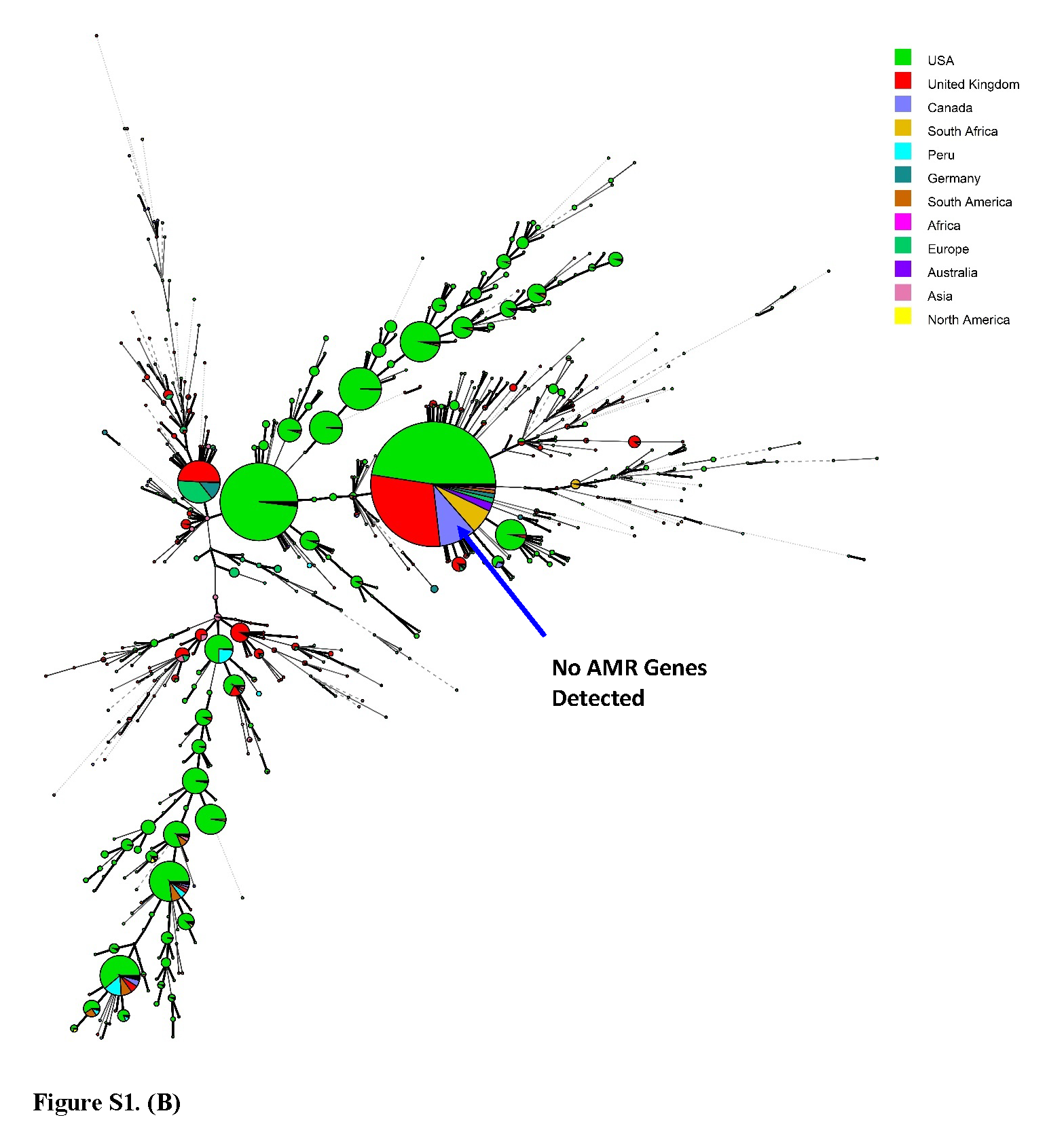

Supplement: Figure S2A — Minimum spanning tree using a composite data set of polymorphic characters for the VFs, AMR genes, and plasmid transfer genes, color coded by source. [file aem.01975-25-s0002.tiff]

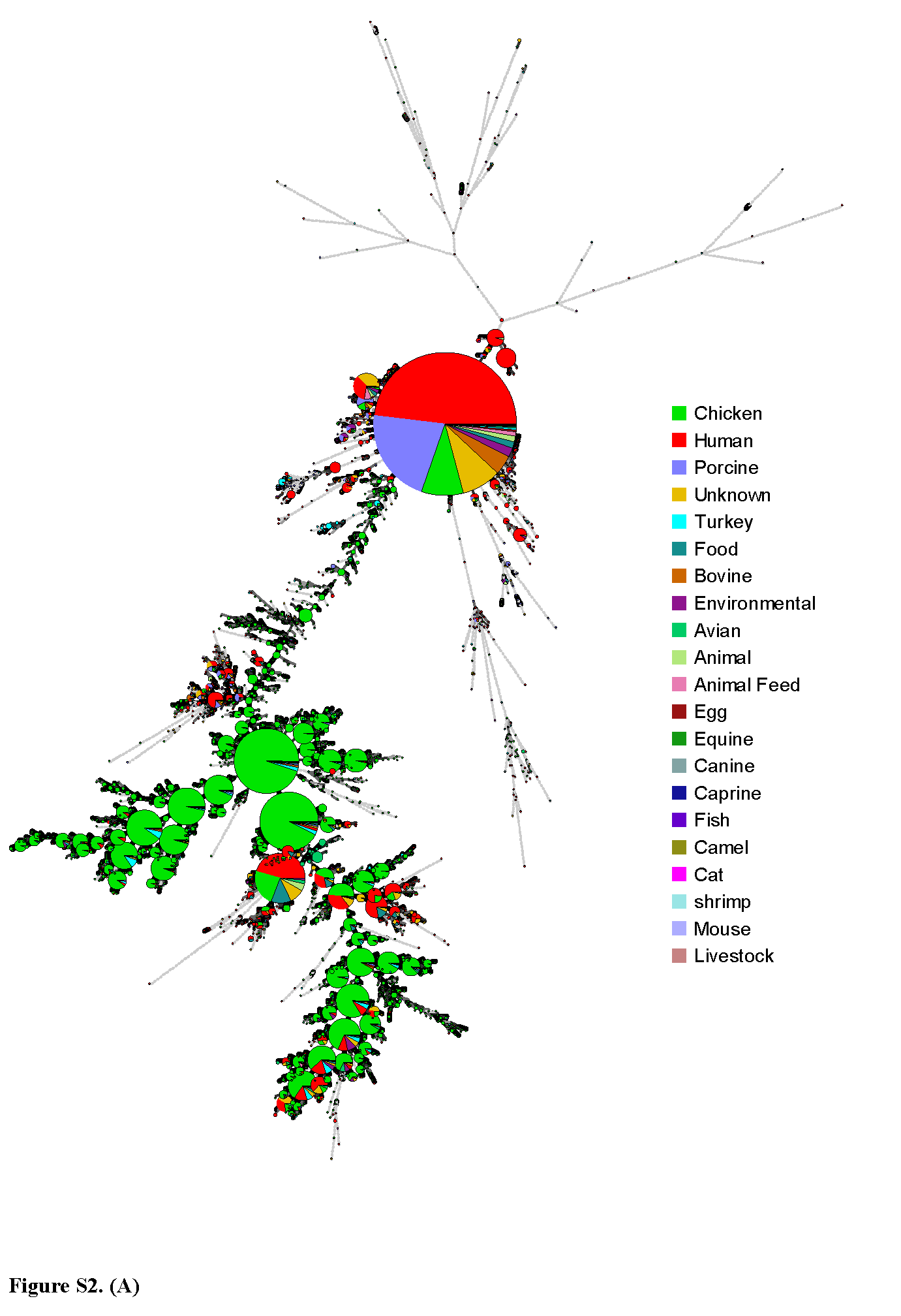

Supplement: Figure S1B — Minimum spanning tree analyses based on the AMR profiles of the isolates, color coded based on locations. [file aem.01975-25-s0003.tiff]

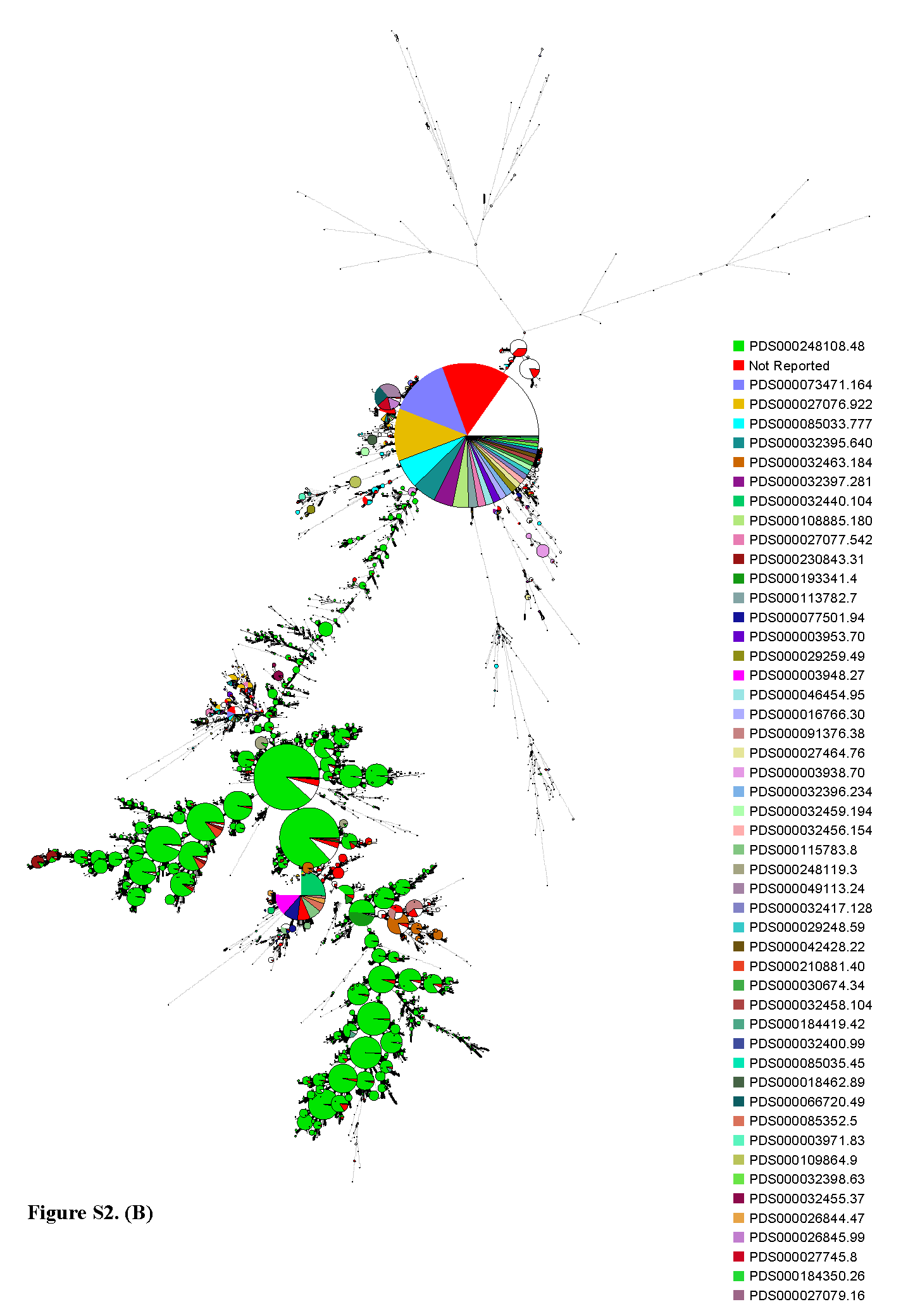

Supplement: Figure S2B — Minimum spanning tree using a composite data set of polymorphic characters for the VFs, AMR genes, and plasmid transfer genes, color coded by SNP type. [file aem.01975-25-s0004.tiff]
