# Supplementary material for: Genomic characterization of antimicrobial resistance and virulence determinants in Salmonella Infantis isolated from human, food, and animal sources
Source: Appl Environ Microbiol. 2026 Mar 23;92(4):e01975-25. doi: 10.1128/aem.01975-25 (PMC13101500; doi:10.1128/aem.01975-25)
Supplement: Supplemental legends — Descriptive legends for Tables S1 to S7 and Fig. S1 and S2. [file aem.01975-25-s0005.docx]

**Supplementary Materials:** Tables S1 to S7 may be accessed at <https://1drv.ms/x/c/eb525f4e9a88a6ff/IQAIVxSywZthTbQiq2BURw-8AV7Zh_pRRCRp08tetjFajfM?e=4aqKb5>.

Table S1: Strains examined in this study, including their metadata; Table S2: AMR gene profiles for all of the strains in the study; Table S3: Plasmid transfer gene profiles for all of the strains in the study; Table S4: Virulence gene profiles for all of the strains in the study; Table S5: Virulence gene profiles of the isolates in the eight largest groups. Table S7: Genome assembly quality metrics for *S*. Infantis isolates analyzed in this study.

Figure S1: Minimum spanning tree analyses based on the AMR profiles of the isolates. The trees are color coded based on years (A) or on locations (B). Figure S2: Minimum spanning tree using a composite dataset of polymorphic characters (defined as being present in between 0.05% and 99.95% of the isolates) for the VFs, AMR genes and plasmid transfer genes to examine profile similarities. Panel A are colored coded by source and panel B by SNP-type.
